# FNDC4 acts as an extracellular factor to promote the invasiveness of hepatocellular carcinoma partly via the PI3K/Akt signalling pathway

**DOI:** 10.1002/cam4.4225

**Published:** 2021-08-21

**Authors:** Baolin Wang, Bowen Zheng, Yao Lu, Deng Huang, Jialong Liu, Juxian Song, Shuguo Zheng

**Affiliations:** ^1^ Institute of Hepatobiliary Surgery Southwest Hospital Third Military Medical University (Army Medical University Chongqing China; ^2^ Department of Hepatobiliary General Hospital of Tibet Military Command Area Tibet China

**Keywords:** extracellular domain, FNDC4, HCC, invasion, PI3K/Akt

## Abstract

FNDC4 is highly homologous to the exercise‐associated myokine FNDC5/irisin, which is highly expressed and promotes the invasion and metastasis of HCC cells. However, the function of FNDC4 remains unknown. Here, we report that FNDC4, an extracellular factor, plays important roles in the invasion and metastasis of HCC. We found that high FNDC4 expression is associated with poor survival in HCC patients and FNDC4 promotes the migration and invasion of HCC cells. Mechanistically, we found that FNDC4 is related to the PI3K/Akt signalling pathway to a certain extent. Specifically, the extracellular domain of FNDC4 acts as an extracellular factor to promote Akt phosphorylation levels in this pathway. These findings reveal that FNDC4 promotes the invasion and metastasis of HCC partly via the PI3K/Akt signalling pathway.

## Graphical abstract

FNDC4 is differentially expressed in human HCC tissues, and high FNDC4 expression is significantly related to the poor prognosis of HCC.

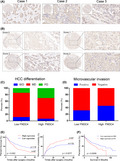

## 1 INTRODUCTION

Hepatocellular carcinoma (HCC) has high invasion and metastasis characteristics that affect the prognosis of patients.^1^ The invasion and metastasis behaviour of tumour cells are responses to their surrounding environment, which depend on various signals of the extracellular matrix and its interaction with neighbouring cells. It is always triggered by extracellular signals from the nearby tumour‐associated matrix.^2^

Fibronectin type III domain‐containing protein 4 (FNDC4), also known as fibronectin type III repeat‐containing protein 1 (Frcp1), belongs to the protein family with members that contain fibronectin type III domains. It is structurally highly conserved, sharing high homology with the exercise‐associated myokine FNDC5/irisin. Its extracellular domain can be cleaved and secreted,^3^ which has the characteristics of extracellular matrix and plays the role of receptor or adhesion molecule. It has been reported that FNDC4, as an anti‐inflammatory factor, reduces the expression of pro‐inflammatory chemokines and significantly reduces the severity of colitis in mice. This anti‐inflammatory role of FNDC4 is also observed in human diseases, such as ulcerative colitis or Chron's disease.^4^ FNDC4 is also a novel adipokine that imitates FNDC5/irisin in human visceral adipocytes to promote fat browning and participate in fat metabolism.^5^ FNDC4 interacts with the Wnt/β‐catenin signalling pathway receptor low‐density lipoprotein receptor‐related protein 6 (LRP6) to affect the differentiation of C2C12 cells.^6^ Moreover, its homologue FNDC5/irisin is highly expressed in hepatocellular carcinoma^7^ and significantly increases cell proliferation, invasion and migration through the PI3K/AKT pathway.^8^ However, the role of FNDC4 in HCC is still unknown.

In this study, we show that FNDC4 plays important roles in the invasion and metastasis of HCC. We found that high FNDC4 expression is associated with poor survival in HCC patients. We have confirmed that FNDC4 promotes the invasion and metastasis of HCC, which is mainly related to the function of the extracellular end of FNDC4. This process is related to the PI3K/Akt signalling pathway. Our results reveal the mechanism by which the extracellular matrix affects the invasion and metastasis of tumour cells by regulating intracellular molecules. This also provides direction to reverse the invasion and metastasis of HCC by regulating the extracellular matrix in the future.

## 2 MATERIALS AND METHODS

### 2.1 Cases and follow‐up

In this study, 205 HCC patients who underwent pathological hepatectomy at Southwest Hospital between January 2010 and October 2015 were recruited by our research team. Patient information (including sex, age, tumour size, TNM stage, HCC differentiation, intrahepatic metastasis, lymph node metastasis, microvascular invasion and other information) was collected by querying patient records and pathological examinations. The patients were followed up until 5 years after surgery, and tumour recurrence and death of the patient were recorded. The study was approved by the Institutional Research Ethics Committee of Southwest Hospital.

### 2.2 Immunohistochemical staining

The pathological tissues of 205 patients were used to produce an HCC tissue microarray. After deparaffinisation and hydration, the antigen was recovered from the microarray by heating in a sodium citrate solution. Then, the microarray was incubated in 3% H_2_O_2_ at room temperature for 10 min and blocked in 10% goat serum for 1 h. The array was incubated with anti‐FNDC4 (1:200, Bioss) antibody at 4°C overnight. An Immunohistochemical Detection Kit (Proteintech) was used to detect the immunohistochemical reaction. After dehydration, the microarray was fixed with neutral resin. The staining intensity and area of the microarray were evaluated by three professional pathologists using CaseViewer software.

### 2.3 Cells and reagents

The hepatoblastoma Hep‐G2 (RRID:CVCL_0027) cell line was obtained from the American Type Culture Collection (ATCC), and the hepatocellular carcinoma Huh‐7 (RRID:CVCL_0336) cell line was obtained from the Japanese Collection of Research Bioresources (JCRB). Both cell lines have been authenticated using STR profiling within the last 3 years. All experiments were performed with mycoplasma‐free cells. The cells were cultured in Dulbecco's modified Eagle's medium (DMEM) (Gibco) supplemented with 10% foetal bovine serum (FBS) (Gibco) at 37°C in a 5% CO_2_ humidified incubator. Referring to the literature,^4^ TGF‐β1 (20 ng/ml) (PeproTech) was used for 24 h to induce endogenous FNDC4 in Huh‐7 cells and to detect the function of endogenous FNDC4.

### 2.4 siRNA transfection and lentivirus infection

MISSION^®^ esiRNA targeting human *FNDC4* and MISSION^®^ siRNA Universal Negative Control were purchased from Sigma‐Aldrich, and Hep‐G2 and Huh‐7 cells were transfected according to the manufacturer's protocol and previous literature.^5^ Overexpression *FNDC4* lentivirus (LV‐FNDC4‐GFP), *FNDC4* extracellular truncated (Δ1‐188) plasmid (pcDNA3.1^+^‐FNDC4‐GFP) and *FNDC4* intracellular truncated (Δ168‐234) plasmid (pcDNA3.1^+^‐FNDC4‐GFP) were constructed by HANBIO Company in Shanghai. Huh‐7 cells were inoculated onto six‐well plates, and lentivirus infections were performed by adding 50 μl of lentivirus (1 × 10^8^ TU/ml) to each well when the cell fusion rate reached 50–70%. After 48–72 h, the *FNDC4* mRNA expression levels were tested experimentally. Plasmids were transfected using Lipofectamine™ 3000 (Invitrogen) according to the manufacturer's protocol.

### 2.5 Quantitative real‐time PCR (qRT‐PCR)

Total RNA was extracted with TRIzol (Beyotime). qRT‐PCR was performed using a Custom Gene qRT‐PCR Quantitation Kit (GenePharma) according to the manufacturer's protocol. This study used the following PCR primers: *FNDC4*‐F: 5′GAGAGCGGCCGCTCGACCTCCCTCTCCTGTG3′; *FNDC4*‐R: 5′GAGAGAATTCATTCCCCTGTCTGCAATGGC3′; *GAPDH*‐F: 5′AGGGGCCATCCACAGTCTTC3′; *GAPDH*‐R: 5′AGAAGGCTGGGGCTCATTTG3′.

### 2.6 Dot blotting

Cell lysates were extracted from each incubated cell culture, and then the corresponding filtered medium was added to the lysates. The mixtures were spotted on a nitrocellulose membrane. The nitrocellulose membranes were blocked with 5% skimmed milk for 2 h at room temperature and incubated with FNDC4 antibody (1:1000, Sigma‐Aldrich) or GAPDH (1:5000, Proteintech) at 4°C overnight. After washing with TBST, the membranes were incubated with a homologous HRP‐conjugated secondary antibody at room temperature for 1 h. Finally, the membranes were detected using a gel imaging system (Vilber, France) with Clarity Western ECL Substrate (Bio‐Rad).

### 2.7 Wound healing assay

The HCC cells were cultivated on a six‐well plate. The scratch was formed by drawing a straight line on the cell surface with a 10 μl pipette tip when the cell confluence reached 100%. Then, the cells were cultured with serum‐free DMEM in a humidified incubator with 5% CO2 at 37°C for 24 h. An inverted microscope was used to monitor cell migration across the scratch line.

### 2.8 Invasion assays

Transwell chambers (8 μm, 24‐well format) (Millipore) were covered with 30 μl of basement membrane Matrigel (diluted 1:6 in DMEM) (Corning Life Sciences) in an incubator at 37°C for 5 h. Then, 200 μl of serum‐free DMEM containing 5 × 10^4^ cells was added to the upper chamber, while 800 μl of DMEM containing 10% FBS was added to the lower wells. After incubation at 37°C for 36 h, the chamber was fixed with 4% paraformaldehyde and stained with crystal violet (Beyotime). After washing with PBS, the Matrigel and cells in the upper chamber were gently removed with a cotton swab. The invaded cells were photographed and counted in three randomly selected 200× fields under a microscope.

### 2.9 Mass spectrometry analysis and bioinformatic analysis

Mass spectrometry (MS) analysis was performed on the lysates of Huh‐7‐overexpressing FNDC4 and normal Huh‐7 cells. MS was performed on a Q Exactive mass spectrometer (Thermo Scientific) that was coupled to Easy nLC (Thermo Scientific). The MS raw data for each sample were combined and searched using MaxQuant 1.5.3.17 software for identification and a quantitation analysis. The protein sequences of the selected differentially expressed proteins were locally searched using NCBI BLAST client software and InterProScan to find homologous sequences. Then, gene ontology (GO) terms were mapped, and sequences were annotated using the software program Blast2GO. Following annotation steps, the studied proteins were BLASTed against the online Kyoto Encyclopedia of Genes and Genomes (KEGG) database (http://geneontology.org/) to retrieve their KEGG orthology identifications and were subsequently mapped to KEGG pathways.

### 2.10 Media transfer culture assays

Huh‐7 and FNDC4‐overexpressing Huh‐7 cells were seeded on a 6‐well plate and cultured at 37°C in a humidified incubator with 5% CO_2_ for 48 h. Huh‐7 cell medium was removed, and FNDC4‐overexpressing Huh‐7 cell medium (filtered) was added to the culture. After 12 h, the medium was repeatedly added 1 or 2 times and then cultured at 37°C in 5% CO2 for 24 h. The cell lysates were extracted for Western blot analysis.

### 2.11 Western blot analysis

HCC cells were lysed with RIPA buffer (Beyotime) containing protease inhibitors and/or phosphatase inhibitors (Beyotime) for 20 min on ice. The lysate was centrifuged at 12000 g for 10 min at 4°C, and the supernatant was collected. The concentration of the supernatant was measured by a BCA Protein Assay Kit (Beyotime). After mixing with loading buffer, the proteins were denatured at 100°C in a metal bath for 10 min. The proteins were separated and then transferred to PVDF membranes (Millipore). After blocking with 5% skimmed milk for 2 h, the membrane was incubated with antibodies (pAkt: 1:2000, Proteintech; GAPDH: 1:10000, Proteintech) overnight at 4°C. The PVDF membrane was blocked with 5% skimmed milk for 2 h at room temperature and incubated with antibodies (pAkt: 1:2000, Proteintech; GAPDH: 1:10000, Proteintech) overnight at 4°C. After washing with TBS, the membranes were incubated with a homologous HRP‐conjugated secondary antibody at room temperature for 1 h. Finally, the membranes were detected using a gel imaging system (Vilber, France) with Clarity Western ECL Substrate (Bio‐Rad).

### 2.12 Statistical analysis

The statistical analysis was performed with Prism 6 (GraphPad). Continuous variables are expressed as the means ± standard deviation and are compared using independent sample *t*‐tests. Categorical variables are compared using chi‐square analysis or Fisher's exact test. All statistical tests were two‐way. *p* < 0.05 was considered statistically significant.

## 3 RESULTS

### 3.1 High FNDC4 expression indicates high invasion and poor prognosis in HCC patients

**FIGURE 1:**
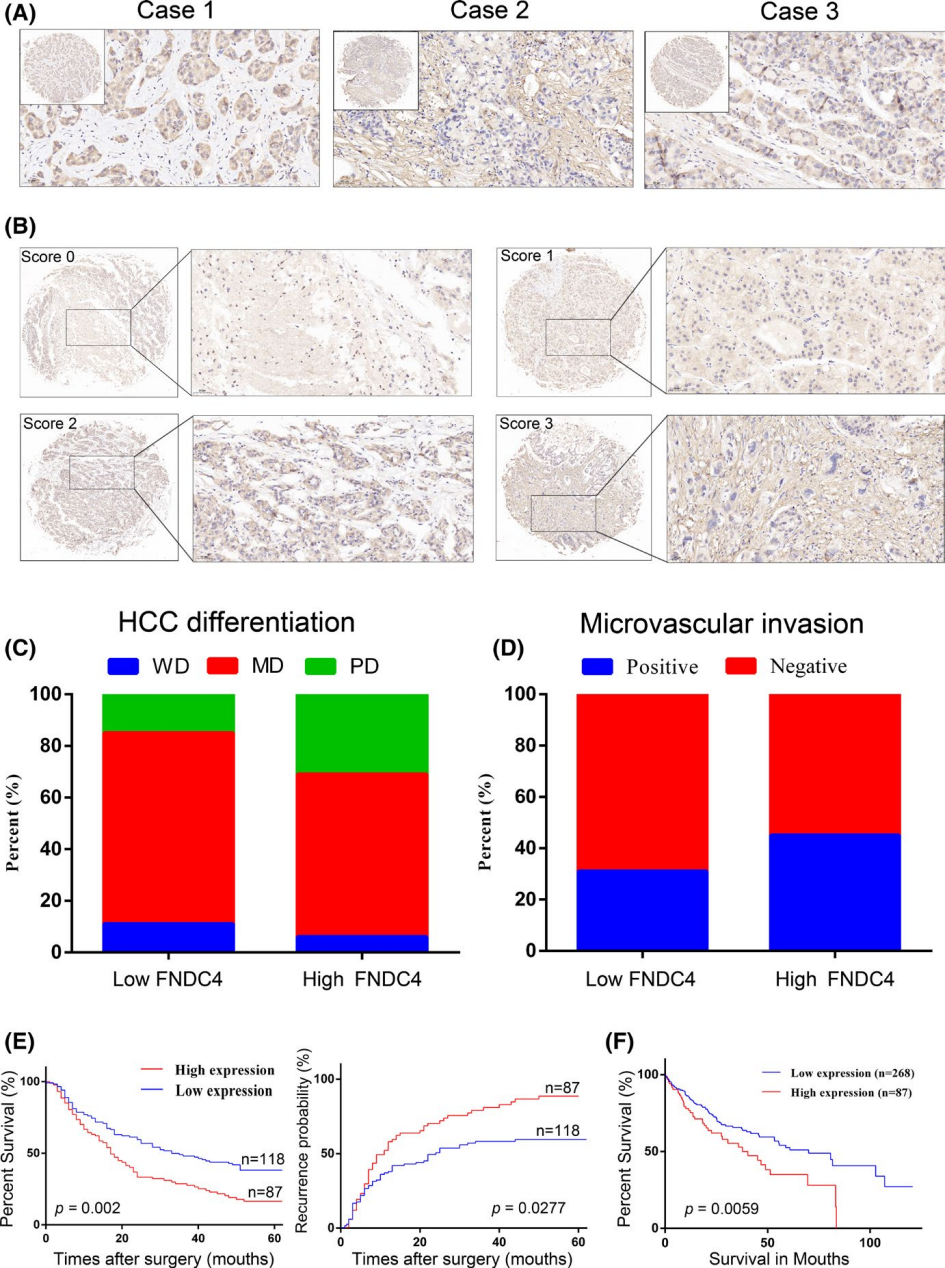
High FNDC4 expression indicates high invasion and poor prognosis in HCC patients. (A) The expression localisation of FNDC4 in IHC in human HCC tissues. In Case 1, FNDC4 expression was mainly in the cytoplasm; in Case 2, FNDC4 expression was mainly in the extracellular matrix; and in Case 3, FNDC4 expression was in both the cytoplasm and the extracellular matrix. (B) Score based on the intensity and area of IHC staining of FNDC4 in HCC. Scores 0–1 represent low expression of FNDC4, while scores 2–3 represent high expression of FNDC4. (C‐D) The expression of FNDC4 in 205 HCC samples was related to HCC differentiation and microvascular invasion. The high expression of FNDC4 implies a higher proportion of poor differentiation and a higher proportion of microvascular invasion (see Table 1, *p* < 0.01). (E) The relationship between the expression of FNDC4 and the survival rate and recurrence rate after surgery in 205 HCC patients. High expression implies a shorter survival rate and higher recurrence rate after surgery (*p* < 0.05). (F) The relationship between the expression of FNDC4 and the survival rate in 355 HCC samples from TCGA database. High FNDC4 expression significantly shortened the survival rate of HCC patients (*p* < 0.01)

To investigate the role of FNDC4 as a secreted protein in HCC, we first analysed its staining localisation in HCC tissues using immunohistochemistry (IHC) (Figure 1A). FNDC4 can be stained in HCC tissues and is mainly expressed in the extracellular matrix, while the cytoplasm and membrane are also distributed. Then, we performed IHC staining on HCC tissues (Figure 1B). We divided scores 0–1 into the low expression group and scores 2–3 into the high expression group. These data indicate that FNDC4 may be related to the occurrence or development of HCC.

To further investigate the correlation of FNDC4 and HCC as well as the prognostic value of FNDC4 in HCC, a tissue microarray‐based IHC study of FNDC4 in 205 HCC tissues with comparable clinicopathological features and complete follow‐up data was performed (Table 1). According to the immunostaining scores, these patients were divided into the high or low FNDC4 expression groups. In 205 HCC patients, the high expression of FNDC4 was significantly correlated with tissue differentiation (*p* = 0.018) (Figure 1C and Table 1) and microvascular invasion (*p* = 0.035) (Figure 1D and Table 1). In addition, the group with high FNDC4 expression was significantly associated with shorter overall survival (*p* = 0.002) and was more likely to have tumour recurrence (*p* = 0.0277) (Figure 1E). Moreover, we analysed the data of 355 HCC samples in The Cancer Genome Atlas (TCGA) database (Figure 1F), and the results were consistent with our results. The high FNDC4 expression group was significantly associated with shorter overall survival (*p* = 0.0059). These data indicate that high FNDC4 expression is significantly related to the poor prognosis of HCC.

**TABLE 1:** Comparison of clinicopathological profiles between low and high FNDC4 expression in HCC patients

Characteristics	NO patients (%)		FNDC4 expression		*p* value	*χ* ^2^
			Low (n = 118)	High (n = 87)		
Age					0.982	0.000
≤50	118	(57.6)	68	50		
>50	87	(42.4)	50	37		
Gender					0.816	0.054
Female	20	(9.8)	12	8		
Male	185	(90.2)	106	79		
Tumour size(cm)					0.723	0.125
≤5	141	(68.8)	80	61		
>5	64	(31.2)	38	26		
Lymph node metastasis					0.147	2.103
Yes	23	(11.2)	10	13		
No	182	(88.8)	108	74		
Intrahepatic metastasis					0.421	0.647
Yes	53	(25.9)	33	20		
No	152	(74.1)	85	67		
TNM stage					0.303	3.637
Stage Ⅰ	53	(25.9)	36	17		
Stage Ⅱ	18	(8.8)	11	7		
Stage Ⅲ	90	(43.9)	47	43		
Stage Ⅳ	44	(21.4)	24	20		
HCC differentiation					**0.018**	**8.063**
WD	18	(8.8)	13	5		
MD	142	(69.3)	87	55		
PD	45	(21.9)	18	27		
Microvascular invasion					**0.035**	**4.426**
Positive	75	(36.6)	36	39		
Negative	130	(63.4)	82	48		
Alpha‐fetoprotein (ng/ml)					0.762	0.092
≤20	52	(25.4)	29	23		
>20	153	(74.6)	89	64		
Liver cirrhosis					0.764	0.091
Yes	132	(64.4)	77	55		
No	73	(35.6)	41	32		
Postoperative recurrence					0.152	2.057
Yes	125	(61.0)	67	58		
No	80	(39.0)	51	29		

Abbreviations: FNDC4, Fibronectin type III domain‐containing protein 4; HCC, hepatocellular carcinoma; MD, moderately differentiated; PD, poorly differentiated; WD, well differentiated.

*p* < 0.05 was considered statistically significant. The significance for bold values is that in 205 HCC patients, the proportion of poor differentiation in tissue with high FNDC4 expression is higher than that with low FNDC4 expression, and the positive rate of microvascular invasion is also higher. These results are statistically significant (Figure 1).

### 3.2 FNDC4 promotes liver cancer cell migration and invasion in vitro

**FIGURE 2:**
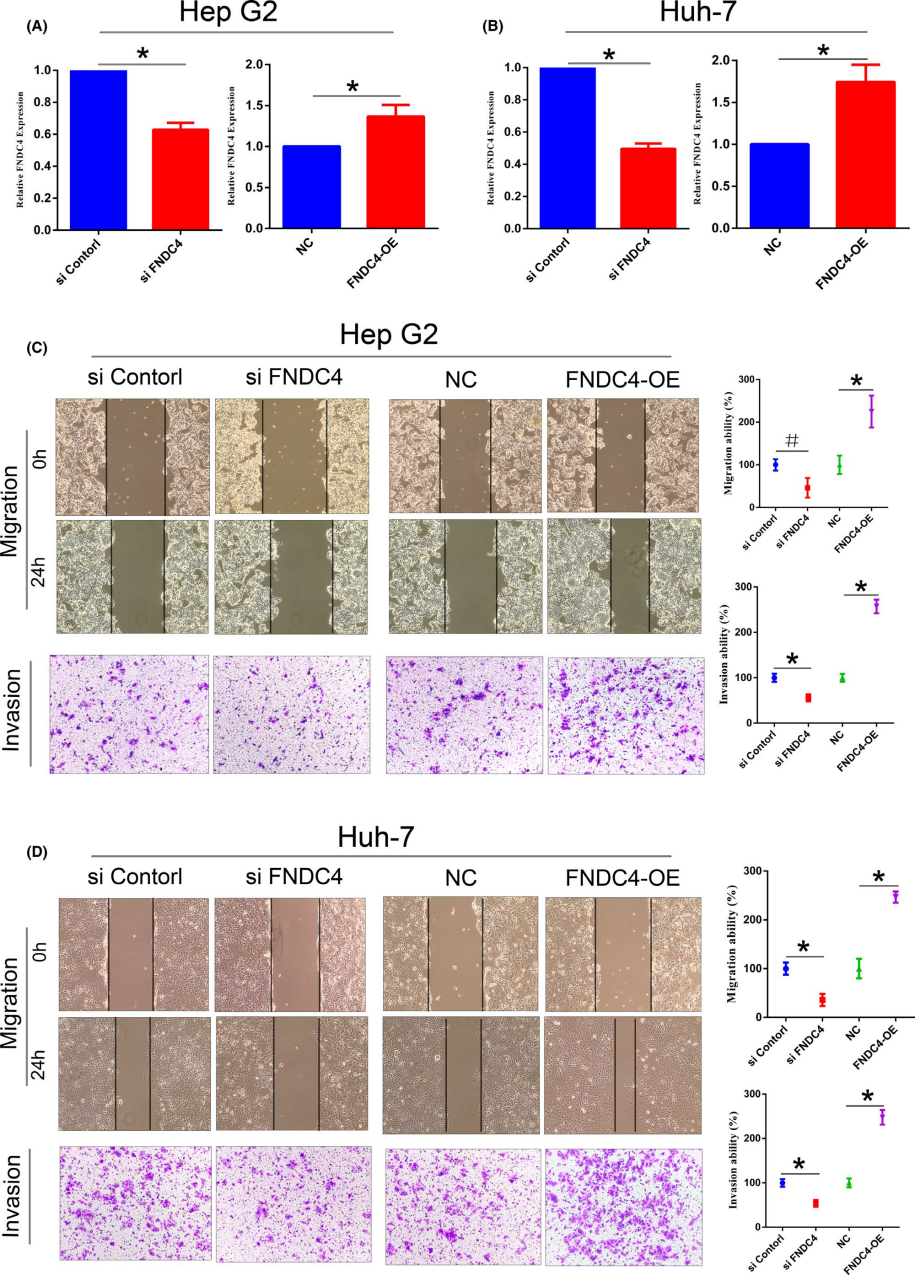
FNDC4 promotes liver cancer cell migration and invasion *in vitro*. (A,B) The mRNA expression level of FNDC4 in Hep‐G2 and Huh‐7 cells after transfection with esiRNA FNDC4 and FNDC4 overexpression lentiviruses, respectively. The FNDC4 esiRNA interfered with the mRNA expression of the two cell lines, while the FNDC4 overexpression lentivirus increased mRNA expression. The FNDC4 mRNA level of Huh‐7 cells was more susceptible to interference and overexpression. (C‐D) The migration and invasion of Hep‐G2 and Huh‐7 cells after interference and overexpression of FNDC4. After FNDC4 was knocked down, the migration rate of both cell lines decreased at 24 h, and the number of invasive cells decreased at 36 h. After overexpression of FNDC4, the results were opposite, and the cell migration and invasion rate increased by nearly 2‐fold (200× fields under a microscope)

To evaluate the effects of FNDC4 on liver cancer cell migration and invasion, we conducted *in vitro* cell assays. First, MISSION^®^ esiRNA targeting human *FNDC4* was used to interfere with Hep‐G2 and Huh‐7 cells, and MISSION^®^ siRNA Universal Negative Control was performed in the same way. The interference of *FNDC4* mRNA reached approximately 40% and 50% in the Hep‐G2 and Huh‐7 cells, respectively (Figure 2A,B). Using lentivirus to overexpress *FNDC4* in Hep‐G2 and Huh‐7 cells, *FNDC4* mRNA levels were upregulated by approximately 140% and 170%, respectively (Figure 2A,B). We found that Hep‐G2 and Huh‐7 cells transfected with esiRNA *FNDC4* showed significantly reduced their migration and invasion *in vitro* (Figure 2C,D), while the migration and invasion of *FNDC4*‐overexpressing cells increased significantly (Figure 2C,D). Notably, Huh‐7 cells show more obvious changes in interference and overexpression systems; therefore, we chose Huh‐7 cells for further testing.

**FIGURE 3:**
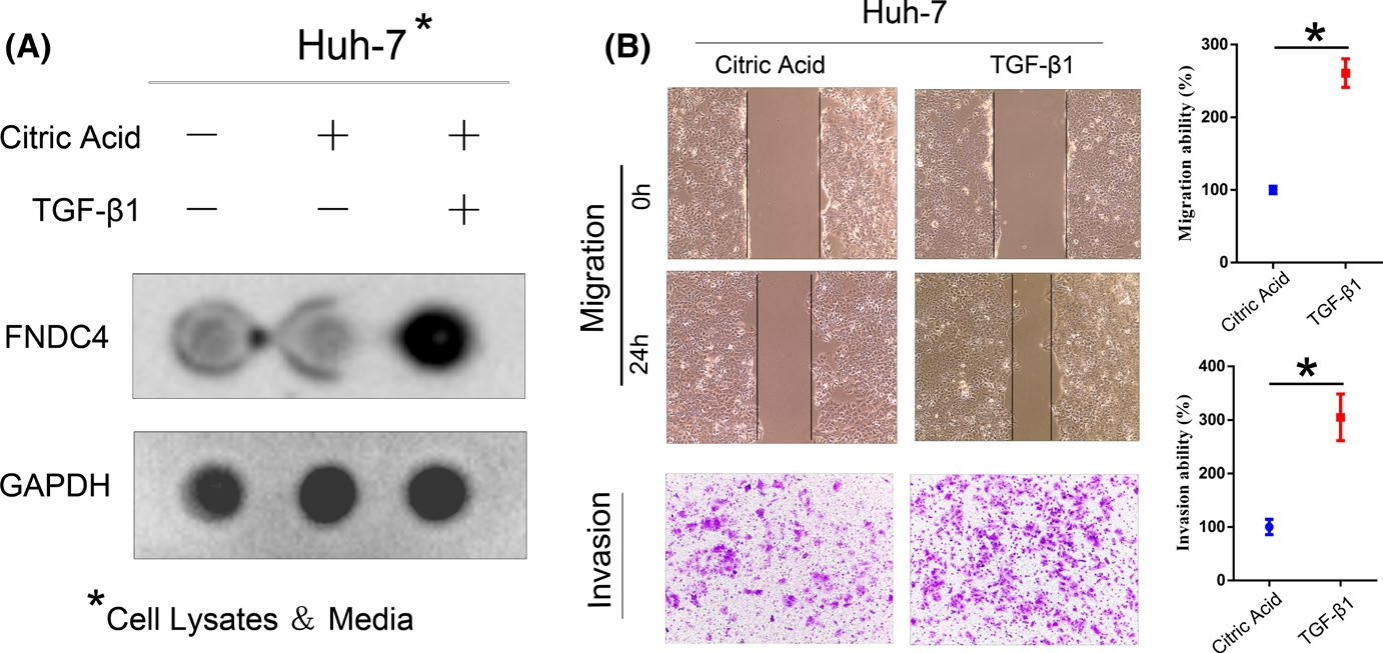
Increased endogenous expression of FNDC4 after treatment with TGF‐β1 also promotes HCC cell migration and invasion *in vitro*. (A) After treatment with TGF‐β1, the expression of FNDC4 increased in Huh‐7 cells. Referring to the literature,^4^ after treating Huh‐7 cells with TGF‐β1 (20 ng/ml) (diluted with citric acid) for 24 h, the cell lysate and culture medium (after filtration) were collected for dot blot assay. The expression level of FNDC4 in Huh‐7 cells treated with TGF‐β1 was significantly higher than that in untreated cells treated with citric acid. (B) The migration and invasion of Huh‐7 cells after treatment with TGF‐β1. After TGF‐β1 treatment, the cell migration rate of Huh‐7 cells increased at 24 h, and the number of invasive cells increased by nearly 3 times at 36 h (200× fields under a microscope)

To confirm these effects of FNDC4, we referred to Bosma, M^4^, who reported that TGF‐β1 stimulates epithelial cells to increase the expression of FNDC4. Similarly, after Huh‐7 cells were used for TGF‐β1 stimulation assays (20 ng/ml), endogenous FNDC4 increased significantly in the cells and their culture media (Figure 3A). Cells treated with TGF‐β1 significantly increased migration and invasion (Figure 3B). We also ruled out the effect of proliferation on cell migration because the increase in proliferation rate was too small (10%–20%) to indicate a significant increase in cell migration (results not shown). In summary, both *FNDC4* overexpression by lentiviral transfection and endogenous *FNDC4* induction by TGF‐β1 promote the invasion and metastasis of liver cancer. These data indicate that FNDC4 is closely related to the migration and invasion of liver cancer cells.

### 3.3 FNDC4 regulates PI3K/Akt pathway in HCC

**FIGURE 4:**
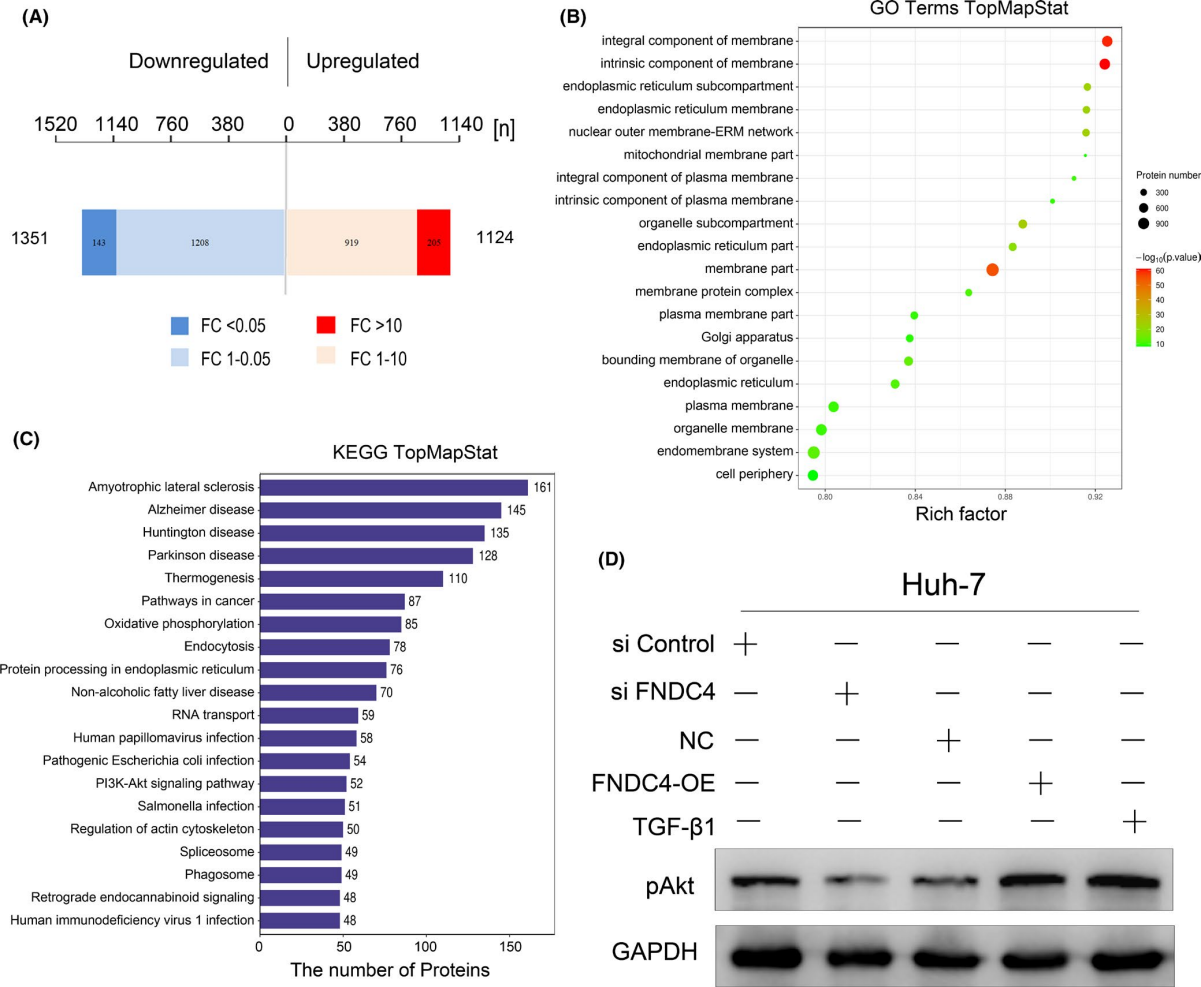
FNDC4 regulates the PI3K/Akt pathway to a certain extent in HCC cells. The cell lysates of Huh‐7 cells overexpressing FNDC4 and control Huh‐7 cells were analysed by mass spectrometry (PRIDE database:PXD025623). (A) Comparing the mass spectrometry results, in FNDC4‐overexpressing Huh‐7 cells, 205 proteins were significantly upregulated compared with NC cells, and 143 proteins were significantly do wnregulated.(B) Gene ontology (GO) terms were mapped and annotated. The upregulated and downregulated proteins were mainly related to cell membrane components. (C) Their KEGG orthology identifications were mapped to pathways in KEGG. The overexpression of FNDC4 may be related to amyotrophic lateral sclerosis, Alzheimer's disease, Huntington's disease, Parkinson's disease, non‐alcoholic fatty liver and cancer pathways and mainly affects the PI3K/Akt signalling pathway. (D) Through Western blot assay, the Akt phosphorylation level of Huh‐7 cells overexpressing FNDC4 and treated with TGF‐β1 was significantly higher than that of control cells

To clarify the molecular mechanism by which FNDC4 promotes HCC invasion, we performed mass spectrometry analysis on FNDC4‐overexpressing cell lysates and control cell lysates. In FNDC4‐overexpressing cells, 1124 proteins were upregulated, and 1351 proteins were downregulated (Figure 4A). Then, Gene Ontology (GO) terms were mapped and confirmed that FNDC4 mainly upregulated membrane‐associated proteins (Figure 4B, PRIDE database:PXD025623). Through KEGG pathway analysis, it was confirmed that FNDC4 may be related to amyotrophic lateral sclerosis, Alzheimer's disease, Huntington's disease, Parkinson's disease, non‐alcoholic fatty liver and cancer pathways and mainly affects the PI3K/Akt signalling pathway (Figure 4C, PRIDE database:PXD025623).

To confirm the regulation of the PI3K/Akt pathway by FNDC4, we verified that siRNA FNDC4 significantly reduced Akt phosphorylation levels, while overexpression of FNDC4 by lentiviral transfection or induction of endogenous FNDC4 by TGF‐β1 increased Akt phosphorylation levels (Figure 4D). Endogenous FNDC4 induced by TGF‐β1 increased more significantly. These data confirm that FNDC4 regulates the PI3K/Akt pathway to a certain extent in HCC cells, which in turn affects the migration and invasion of HCC cells.

### 3.4 The extracellular domain of FNDC4 affects the PI3K/Akt pathway

**FIGURE 5:**
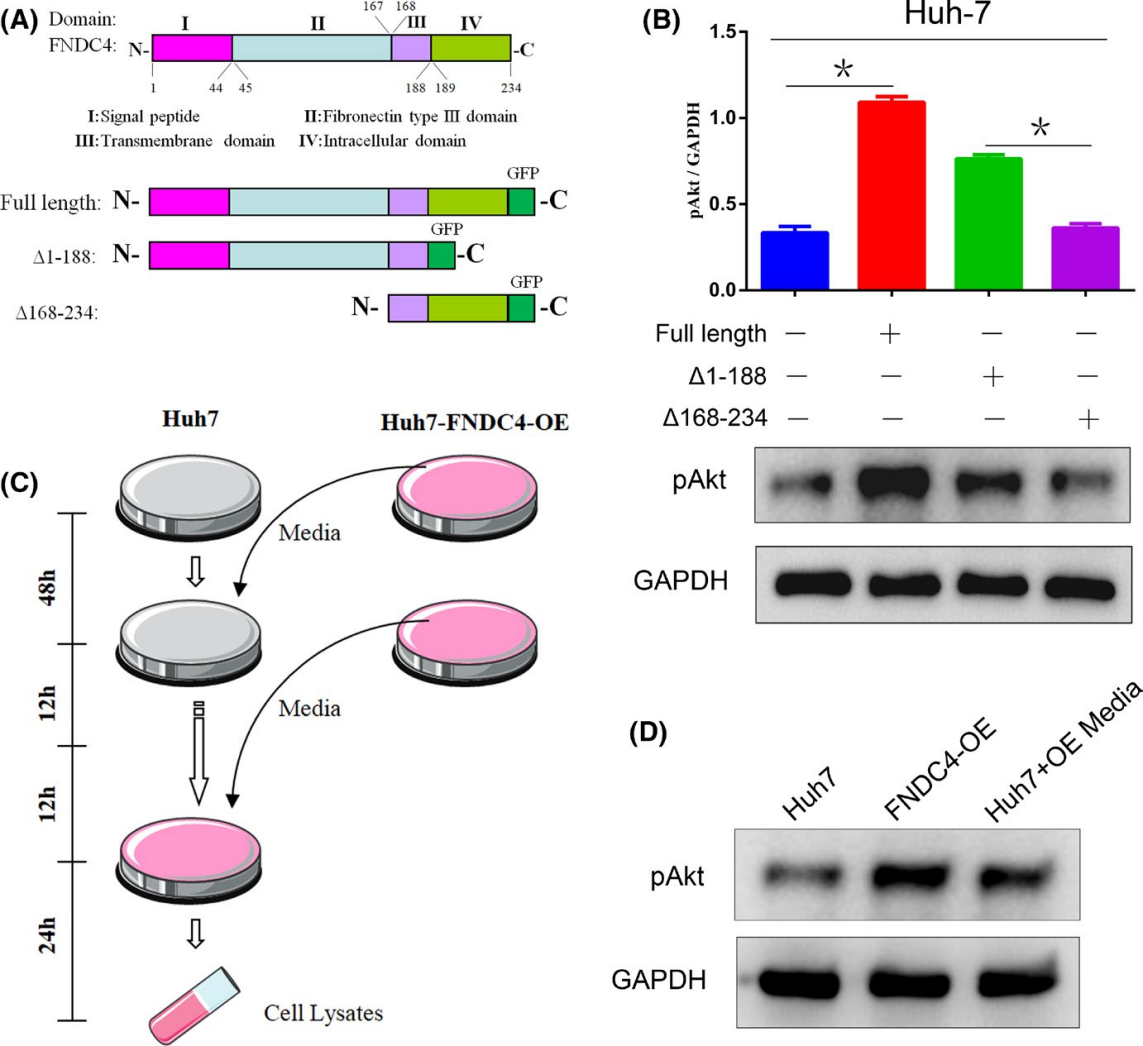
The extracellular domain of FNDC4 affects the PI3K/Akt pathway. (A) Schematic diagram of each domain of FNDC4 protein. Schematic diagram of full‐length FNDC4, truncated extracellular domain (Δ1‐188) and truncated intracellular domain (Δ168‐234). (B) Each truncated FNDC4 affected the phosphorylation level of Akt after transfection into Huh‐7 cells. The truncated Δ1‐188 mutant showed the function of overexpressing full‐length FNDC4, while the truncated Δ168‐234 mutant failed to affect the level of Akt phosphorylation. (C) Schematic diagram of the media transfer assays. Normal Huh‐7 cell medium was removed, and FNDC4‐overexpressing Huh‐7 cell medium (filtered) was added to the culture. After 12 h, the medium was repeatedly added 1 or 2 times to continue cultivation. (D) After transferring FNDC4‐overexpressing Huh‐7 cell medium to normal Huh‐7 cells, the Akt phosphorylation level of the latter was significantly increased, as shown by Western blot assay and was close to the level of the former

To further evaluate the mechanism by which FNDC4 regulates the PI3K/Akt pathway in HCC cells, Huh‐7 cells were transfected with full‐length *FNDC4*, extracellular truncated *FNDC4* (Δ1‐188) and intracellular truncated *FNDC4* (Δ168‐234) plasmids (Figure 5A). Cells harbouring full‐length and extracellular truncated *FNDC4* showed significantly increased Akt phosphorylation levels, while intracellular truncated FNDC4 showed no clear effect on cells (Figure 5B). Therefore, we determined that the extracellular domain of FNDC4 played a role in the PI3K/Akt pathway.

To clarify these conclusions, we designed media transfer culture assays in which FNDC4‐overexpressing Huh‐7 cell medium was added to normal Huh‐7 cells to simulate the role of the FNDC4 extracellular domain (Figure 5C). The Akt phosphorylation levels of normal cells overexpressing FNDC4 in cell medium also increased significantly (Figure 5D). These assays confirmed that the extracellular domain of FNDC4, but not the intracellular domain, regulates the activity of the PI3K/Akt pathway to a certain extent.

## 4 DISCUSSION

HCC metastasis is the main cause of death after surgery.^9^ Similar to other tumour cells, the invasion and metastasis of HCC cells reflect the biological behaviour of cells in which a variety of proteins and molecules in the intracellular and extracellular matrix participate cooperatively. The extracellular matrix can affect the invasion and metastasis of tumour cells.^10^

Fibronectin type III (FNIII) acts as a receptor or adhesion molecule in tumour cells and participates in a variety of biological processes of tumour cells, such as cell migration and differentiation.^11^ FNDC4 belongs to the protein family containing the FNIII domain. In a relatively small but well‐characterised cohort of HCC patients, we detected that high FNDC4 expression in HCC tissues is closely related to the poor prognosis of HCC patients. Certainly, a larger cohort can increase the statistical power and, hence, establish the specific contribution of FNDC4 in the invasion and metastasis of HCC. Wuensch, T et al.^12^ analysed mucosal samples from patients with colorectal cancer (CRC). FNDC4 is highly expressed in CRC (although there is no statistically significant difference). We determined that FNDC4 plays a role in HCC, and then, both gain‐ and loss‐of‐function experiments showed that FNDC4 promotes the migration and invasion of HCC cells. To exclude the influence of external factors, we referred to Bosma, M^4^ who used TGF‐β1 to stimulate HCC cells to increase the endogenous expression of FNDC4, which also increased the invasion and migration of HCC cells. TGF‐β1 stimulation more significantly increased Akt phosphorylation levels. We found that this may be related to the involvement of TGF‐β1 in costimulation, including MMP‐8,^13^ NCX1/TRPC6^14^ and other factors, or that TGF‐β1 may indirectly promote the expression of FNDC4. These assumptions need further research and clarification.

Then, we further studied the molecular mechanism of FNDC4 in HCC cells. Through KEGG pathway analysis, we confirmed that FNDC4 is related to cancer pathways and mainly affects the PI3K/Akt signalling pathway. We have also confirmed that FNDC4 regulates the activity of the PI3K/Akt pathway to a certain extent in HCC cells, and this effect is due to its extracellular domain rather than its intracellular domain. The Arg‐Gly‐Asp (RGD) sequence is a common ligand for integrin receptors, which can confer cell adhesion properties to many proteins.^15^ The molecular structure of FNDC4 contains only the RGD sequence in the FNIII domain. The RGD sequence of FNDC4 interacts with integrin β1 (ITGβ1) to activate focal adhesion kinases p‐FAK, p‐paxillin and vinculin and promote the migration and differentiation of bovine skeletal muscle‐derived cells.^16^ Moreover, FDNC4 can influence the differentiation of C2C12 by activating Wnt/β‐catenin signal transduction. The mechanism is also that the extracellular domain of FNDC4 acts as a receptor‐binding ligand can interact with the Wnt/β‐catenin signalling pathway receptor LRP6.^6^ The RGD sequence of the extracellular domain may also play an important role in this process. Therefore, we speculate that the role of FNDC4 in HCC cells may also be transmitted to intracellular molecules through its RGD sequence and membrane receptor interaction, which will be studied in depth in our next work.

Currently, there are relatively few research reports on FNDC4 in tumours. Because FNDC4 is an extracellular secretory protein, general protein detection methods are inefficient. There have been reports on the application of extracellular matrix proteomics. Moreover, the role of extracellular proteins is not conducive to verification in animal models because it is affected by many factors. In this study, we investigated the effect of the extracellular domain of FNDC4 on HCC by transferring FNDC4‐overexpressing cell medium. This experimental method is not very accurate and difficult to quantify. Nevertheless, it can be clearly confirmed that the extracellular domain of FNDC4 regulates the activation of the PI3K/Akt signalling pathway to a certain extent. This also confirms that the role of FNDC4 in promoting the invasion and metastasis of HCC is partly related to the activity of the PI3K/Akt signalling pathway.

In conclusion, we identified FNDC4 as an important extracellular molecule for HCC cell invasion and metastasis, and its high expression indicates a poor prognosis in HCC patients. The role of FNDC4 in HCC is to regulate the activity of the PI3K/Akt signalling pathway to a certain extent through its extracellular domain. We will further study the molecular mechanism of this pathway. Therefore, designing inhibitors that target the expression of extracellular secreted proteins (e.g. FNDC4) may be a promising method to inhibit HCC invasion and metastasis in the future.

## ETHICS APPROVAL AND CONSENT TO PARTICIPATE

Human HCC tissues samples were from Institute of Hepatobiliary Surgery, Southwest Hospital, Third Military Medical University (Army Medical University). Moreover, written consent was received from each patient. The study was approved by the Institutional Research Ethics Committee of Southwest Hospital.

## CONFLICT OF INTERESTS

The authors declare that no conflicts of interest exist.

## AUTHOR CONTRIBUTIONS

Baolin W. performed experiments, analysed data and wrote the paper; Bowen Z. Yao L. Deng H. Jialong L. and Juxian S. performed some experiments; Shuguo Z. initiated the study, designed experiments and wrote the paper.

## Data Availability

The raw mass spectrometry data generated in this study has been deposited in the PRIDE database the dataset identifier PXD025623. Other data that supports the findings of this study are available from the corresponding author upon request.
